# Viscosity Behavior of P(DAC-AM) with Serial Cationicity and Intrinsic Viscosity in Inorganic Salt Solutions

**DOI:** 10.3390/polym11121944

**Published:** 2019-11-26

**Authors:** Tingting Chen, Xingqin Fu, Luzi Zhang, Yuejun Zhang

**Affiliations:** School of Chemical Engineering, Nanjing University of Science and Technology, Nanjing 210094, China

**Keywords:** P(DAC-AM), intrinsic viscosity, cationicity, salt solution, valence, apparent viscosity, mechanism model

## Abstract

The poly(acryloyloxyethyl trimethyl ammonium chloride–*co*–acrylamide), P(DAC-AM), is a kind of cationic polyelectrolyte usually applied in a solution form, and its performance is affected by its structure and the environment where it is used. In particular, its viscosity properties in salt solutions are directly related to its efficacy in various applications, and the performance is one of the most important solution properties. Therefore, in this paper, the effects of the salt concentration and valence of seven kinds of inorganic salts, NaCl, LiCl, KCl, MgCl_2_, AlCl_3_, Na_2_SO_4_, and Na_3_PO_4_, on the values of apparent viscosity (*η*_a_) of P(DAC-AM) samples with cationicity of 10%, 50%, and 90%, and intrinsic viscosity ([*η*]) of 5, 10, and 15 dL/g were investigated. The *η*_a_ was determined using a rotational viscometer. The interaction mechanism between the polymers and salt ions was also investigated. The results showed that depending on the salt concentration, the *η*_a_ firstly decreased sharply to the inflection point which indicated the minimum volume of the molecule shrinking, and then either maintained the value unchanged or increased. The salt concentration corresponding to the inflection point decreased with the increase of the salt ion valence but with the reduction of the cationicity of the polymer. The *η*_a_ at the inflection point increased as the [*η*] of the polymer grew. This indicated that the salt concentration and the salt ion valence had a notable impact on the stretch of the cationic polymer molecule in the salt solutions. It was discovered that the phenomenon of the increase of the *η*_a_ of P(DAC-AM) samples in the multivalent salt solutions after the inflection point was caused by not only the increase of the *η*_a_ of the complexes formed from the pure salts, but also the viscosity resistance of the charge and volume between the polymer molecules and salt ions, as well as the complexes themselves. The linear relationship between the increased *η*_a_ and the salt concentration, representing the interaction both among the complexes themselves and between the polymer and complexes, was obtained. Furthermore, the interaction model between the salt ions and P(DAC-AM) molecules in a wide range of salt concentrations was illustrated.

## 1. Introduction

Polyelectrolyte is a kind of water-soluble polymer, easily dissolved in the form of macromolecular solution and releasing a large amount of counter ions which makes it become the charged colloidal particle. It can be divided into poly-ampholyte, anionic, and cationic electrolytes depending on the charge property of the colloidal particle. The poly (acryloyloxyethyl trimethyl ammonium chloride–*co*–acrylamide), P(DAC-AM), is the water-soluble polymer with a positive charge, i.e., cationic polyelectrolyte. It is widely applied in various industries, such as biology engineering, environmental protection, medicine, chemical materials, and nanotechnology [1,2]. Generally, P(DAC-AM) is usually applied in the solution forms and its performance is always closely related to their property in the solution state. The solution properties depend on not only the molecular components and unit structure, but also the whole structure size and charge density of the P(DAC-AM) molecule in solution, including molecular weight, molecular weight distribution, cationicity, and so on [3,4]. Therefore, research on the solution properties and the factors on the change of the solution properties are essential for achieving optimum efficiency in developing high-performance formulations with P(DAC-AM) components [5]. Among them, the viscosity property of polyelectrolytes, which is directly related to the various applications and performance, is one of the important solution properties.

At present, there have been many studies reporting on the viscosity property of water-soluble polymer solutions [6,7,8,9]. It is well known that the factors influencing the viscosity property of polymer solutions include, not only the polymer structure itself, but also the salts, pH, surfactants, and so on, in the solutions [10,11,12]. Due to the existence of salts in most polymer solutions when in practical application, the effect of the salts on the viscosity property of the polymer solution is still a topic of interest [8,13]. Since the 1960s, research on the interaction between the polymer and salt ions indicated that the viscosity behavior of the polymer in the simple salt solutions was related to the interaction between the polymer and salt ions when in low concentration. There is, not only the hydrogen-bond interaction, but also the dipole interaction, and the research results are comparable with the existing model of the interaction between inorganic salt ions [14,15]. Until the 1990s, with the increase in the kinds of synthetic and modified polymers and the increase of the valence state of used salt ions, the research on interactions between the polymer and monovalent (e.g. Na^+^, K^+^, Cl^−^, Br^−^, and I^−^) or bivalent (e.g. Mg^2+^, Ca^2+^, and SO_4_^2−^) salt ions was put forward, so that the interaction might be regarded as an electrostatic repulsion interaction. Then a kind of model of this interaction between the polymer and monovalent salt ions was established [8,16,17]. However, in recent years, for the research on the mechanism or model between the polymer and salt ions, not only was the measurement improved, from viscometer to dynamic light scattering, and so on [6,18], but also the objective parameters measured were changed from macroscopic viscosity to microscopic ionic radius [11,19]. Meanwhile, the effect of the trivalent salt ion (Al^3+^) on the viscosity property of polymers in salt solutions was mentioned in reports [13], however, only the model of the interaction between the polymer and monovalent or bivalent salt ions was established [20,21,22]. In addition, the theoretical computations for quantitative analysis of the effect of polymer microstructure on the viscosity property in salt solution was studied, more and more [23].

Since the beginning of the 21st century, there have been reports about the effect of the surfactants on the viscosity property of the P(DAC-AM) solutions [24]. In the recent ten years, the viscosity property of the P(DAC-AM) in the NaCl solution was reported [7,16,25,26]. It was barely used as one of the sample characterization of an individual P(DAC-AM) synthesized, or it was used as a control sample in the characterization of its derivatives. For example, Gemma G. G. [16] used gel permeation chromatography (GPC) to study the effect of changing NaCl salt solution concentration, from 0 to 0.10 mol/L, on the determination accuracy of P(DAC-AM) with four kinds of cationicity (i.e., 0%, 21%, 52%, and 100%) and molecular weight lower than 5 × 10^5^ g/mol. The results showed the viscosity of the polymer decreased with the salt concentration. However, in the research, the molecular weight of the samples and salt concentrations involved were all very low. Huang P. [25] used the P(DAC-AM) as the contrast sample of the CPAM/MMT (polymer of P(DAC-AM) and montmorillonite). The reduced viscosities of the P(DAC-AM) with an intrinsic viscosity ([*η*]) of 7.25 mL/g and CPAM/MMT with an [*η*] from 3.47 to 5.68 mL/g were determined by the Ubbelohde viscometer and the rheological properties of two polymers were determined by rheometer. The results showed the elastic modulus of P(DAC-AM) was larger than CPAM/MMT. This study described the viscosity properties of the polyelectrolyte solutions with different cationicity and molecular weights, but their interaction with salt was not mentioned. 

However, for the P(DAC-AM) samples with serial cationicity and molecular weights (i.e., [*η*]), there had been rare studies systematically reporting their viscosity properties in different kinds of salt solutions. The reasons for the above were as follows. On the one hand, due to the limitation of the sample kinds commercially available or the synthetic level in the laboratory, not only were the kinds and number of polymer P(DAC-AM) samples used relatively small, but also the molecular weights of the polymers used were often limited to lower than 1 × 10^6^ g/mol. So, unfortunately, there were no studies reporting the viscosity property of salt solutions of P(DAC-AM) with higher molecular weights, until now. On the other hand, multivalent salt solutions were particularly less studied in sufficiently high concentrations, as the salt solutions used were mainly focused on the monovalent and divalent salt solutions. So the research on trivalent salt solutions has been never reported, until now. Due to the existence of the above difficulties, the motivation, both in the deep understanding of the viscosity property of salt solutions of P(DAC-AM), and in the comprehensively theoretical guidance of the viscosity property in practical applications were greatly required. Therefore, it was necessary to study the viscosity property of salt solutions of P(DAC-AM) with serially controlled cationicity and [*η*] [22].

The values of apparent viscosity (*η*_a_) of polymers in salt solutions meant that the summation of the viscosity resistance existed among the internal components when the fluid was flowing [27]. So, in this work, the *η*_a_ of the P(DAC-AM) samples with serial cationicity and [*η*] in different salt solutions would be systematically determined by a viscometer to investigate the change tendency, possible regulation, and mechanism in an inorganic salt solution, such as NaCl, LiCl, KCl, MgCl_2_, AlCl_3_, Na_2_SO_4,_ or Na_3_PO_4_ solutions. In particular, the *η*_a_ and its change regulation and mechanism of P(DAC-AM) samples in the divalent and trivalent salt solutions were focused on and compared with the situations of the monovalent salt solution. Eventually, the aggregation and interaction model describing the viscosity property of P(DAC-AM) molecules in salt solutions would be proposed. Therefore, the interaction regulation and mechanism between the different salt ions and the polyelectrolyte P(DAC-AM) molecules with serially controlled caitonicity and [*η*] could be obtained. The results could provide the theoretical and experimental basement for the research on other properties, theoretical computation of their salt solutions, and the practical application of P(DAC-AM) samples with serial cationicity and [*η*].

## 2. Experimental

### 2.1. Samples

The P(DAC-AM) colloid samples with serially controlled cationicity and intrinsic viscosity ([*η*]) were synthesized in the laboratory [28] and they were refined to a dry powder by recrystallization in a mixed solvent of ethanol and acetone. The cationicity of the initial molar fraction of acryloyloxyethyl trimethyl ammonium chloride (DAC) in the copolymerization, was 10%, 50%, and 90%, and the ranges of [*η*] (of samples dissolved in 1 M NaCl were determined at 30 ± 0.1 °C by using an Ubbelohde viscometer (Nanjing, China), and [*η*] could be calculated based on this, and was used to often represent molecular weight [3]) were 4.90–5.10, 9.90–10.10, and 14.90–15.10 dL/g.

The representative monovalent, divalent, and trivalent salts were selected as follows: lithium chloride (LiCl, AR), sodium chloride (NaCl, AR), potassium chloride (KCl, AR), magnesium chloride hexahydrate (MgCl_2_·6H_2_O, AR), aluminum chloride anhydrous (AlCl_3_, AR), sodium sulfate (Na_2_SO_4_, AR), and sodium phosphate (Na_3_PO_4_·12H_2_O, AR) from Chengdu Cologne Chemical Co. Ltd. (Chengdu, China) or Shanghai Aladdin Bio-Chem Technology Co. Ltd. (Shanghai, China) were used as purchased.

### 2.2. Measurement of Apparent Viscosity

The values of apparent viscosity (*η*_a_) of salt solutions of P(DAC-AM) samples with serial cationicity and [*η*] were measured using a Brookfield DV2T rotational viscometer (MA, US) with a #1 rotor at 200 rpm, and at a temperature of 25 °C.

### 2.3. Methods

(1) Determination of viscosity property of polymer in salt solutions

In order to study the effect of salt concentration on the *η*_a_ of P(DAC-AM) samples with serial cationicity and [*η*] in inorganic salt solutions, such as NaCl, LiCl, KCl, MgCl_2_, AlCl_3_, Na_2_SO_4,_ and Na_3_PO_4_ solutions, the *η*_a_ of P(DAC-AM) samples with cationicity of 10%, 50%, and 90%, and [*η*] of 5, 10, and 15 dL/g were determined using a Brookfield DV2T rotational viscometer in different salt solutions with concentrations from 0 to 1.000 mol/L [16]. 

Firstly, the P(DAC-AM) sample with specific cationicity and [*η*] was weighed and dissolved in distilled water to achieve a mass concentration of 10 g/L. Then, it was added into the pure salt solutions from 0 to 1.000 mol/L, to dilute the mass concentration of the polymer to 1 g/L [29], a commonly used concentration of this polymer. After that, the *η*_a_ of salt solutions of the P(DAC-AM) sample was determined using a Brookfield DV2T rotational viscometer, according to the procedures in Section 2.2.

(2) Assessment of the influencing factors on the viscosity property.

With the change of different salt solution concentrations and salt valence, the change tendency and possible regulation of the *η*_a_ of P(DAC-AM) samples with different cationicity and [*η*] in the salt solutions were investigated, specially emphasizing on the possible abrupt inflection point or the lowest value [16], and their influence factors. At the same time, the difference of change tendency and possible regulation of *η*_a_ of P(DAC-AM) samples in the monovalent and multivalent salt solutions was compared. With that, we attempted to obtain a better description of their interaction regulation, and even the mechanism.

(3) The interaction mechanism model of salt ions and polyelectrolyte molecules.

Based on the literature [20,22] and the above results, we studied the mutual function mechanism between the polymer molecules and salt ions in different salt conditions, and meanwhile, the aggregation model of interaction between polyelectrolyte P(DAC-AM) molecules and salt ions was developed.

## 3. Results and Discussion

### 3.1. The General Regulations of η_a_ of P(DAC-AM) Samples in the Salt Solutions

According to the methods in Section 2.3, the effect of salt concentration on the values of apparent viscosity (*η*_a_) of P(DAC-AM) with cationicity of 10%, 50%, and 90% and intrinsic viscosity ([*η*]) of 5, 10, and 15 dL/g under the concentration of 1 g/L in the inorganic salt solutions was investigated using a rotational viscometer. 

#### 3.1.1. The Change Tendency of *η*_a_ of P(DAC-AM) Samples with Salt Concentration in the Monovalent Salt Solutions

**Figure 1:**
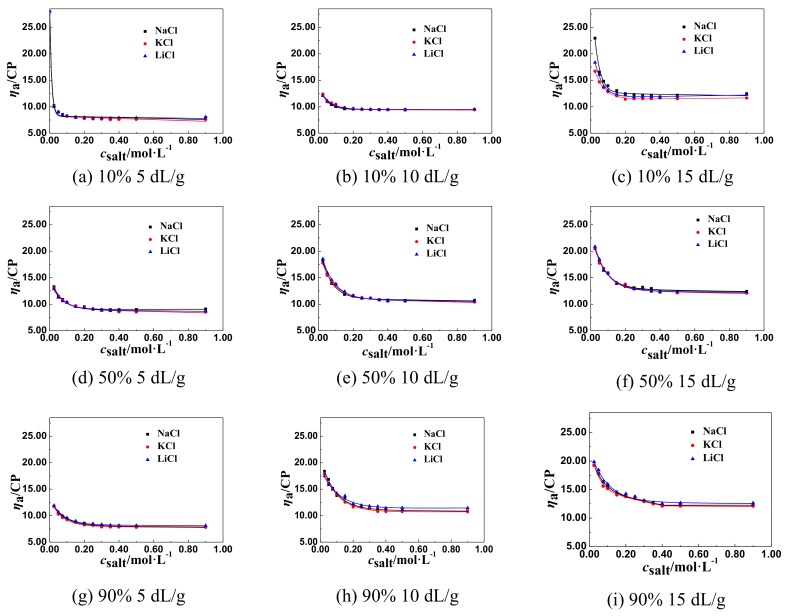
The effect of monovalent salt concentration on the *η*_a_. The unit for the y-axis is mPa·s (CP).

The effect of monovalent salts, like NaCl, LiCl, and KCl, on the *η*_a_ of the solutions of P(DAC-AM) samples with serial cationicity and [*η*] was shown in Figure 1.

From Figure 1, it was easily found that all *η*_a_ of P(DAC-AM) firstly decreased sharply to the lowest value with the increase of the salt concentration of LiCl, NaCl, and KCl. When the inorganic monovalent salts were added into the polyelectrolyte solutions, the supplied counter ions screened the charge of the polymer colloid, and so the intramolecular repulsion in the polyelectrolytes was weakened, and the molecules were shrunken as the curl and the *η*_a_ of solutions decreased to an unshrinkable state representing the lowest value of *η*_a_ of solutions [30,31]. Moreover, the lowest value of *η*_a_ of a P(DAC-AM) sample in a salt solution was almost unchanged within a certain range with the salt concentration increasing further [32,33]. However, the cationicity and [*η*] of P(DAC-AM) samples and the monovalent salt ions impacted differently on the lowest value of *η*_a_.

#### 3.1.2. The Change Tendency of *η*_a_ of P(DAC-AM) Samples with Salt Concentration in the Multivalent Salt Solutions

##### The *η*_a_ of P(DAC-AM) Samples in the Multivalent Salt Solutions of Different Cations

**Figure 2:**
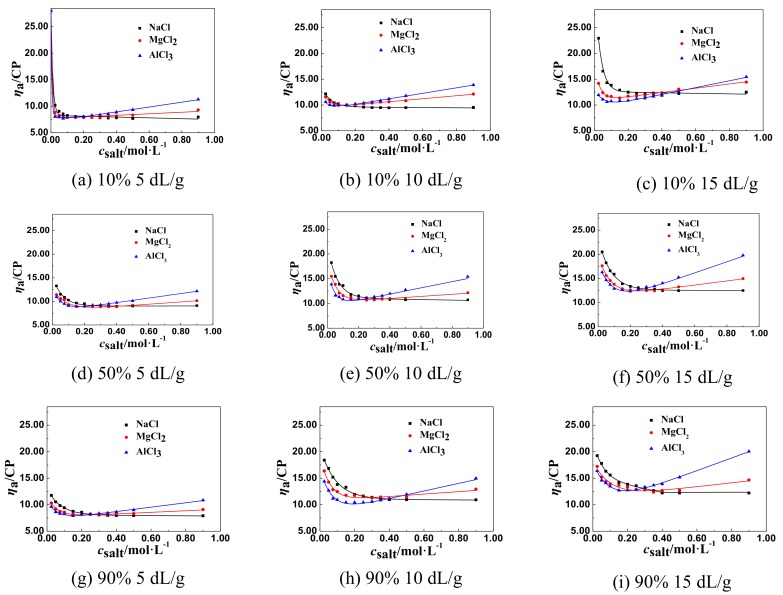
The effect of salt concentration of multivalent salts of different cations on the *η*_a._

The effect of NaCl and multivalent salts of different cations, such as MgCl_2_ and AlCl_3_, on the *η*_a_ of P(DAC-AM) samples with serial cationicity and [*η*], was shown in Figure 2. 

From Figure 2, we found that the *η*_a_ of P(DAC-AM) samples firstly decreased sharply to the lowest value, but then increased with the concentration of multivalent salts of different cations, such as MgCl_2_ and AlCl_3_. Then, before the lowest value, the change of the *η*_a_ of P(DAC-AM) samples to the lowest value was accelerated by increasing the valence of the multivalent salts of different cations. After the lowest value, the rise rate of *η*_a_ of P(DAC-AM) samples was enlarged with the increase of the valence of the multivalent salts of different cations.

##### The *η*_a_ of P(DAC-AM) Samples in the Multivalent Salt Solutions of Different Anions

**Figure 3:**
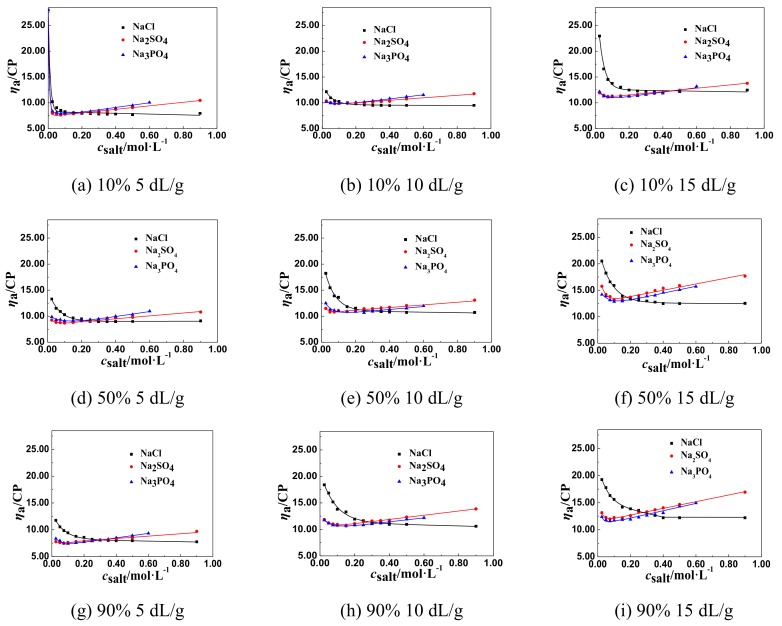
The effect of salt concentration of multivalent salts of different anions on the *η*_a._

The effect of NaCl and multivalent salts of different anions, such as Na_2_SO_4_ and Na_3_PO_4_, on the *η*_a_ of P(DAC-AM) samples with serial cationicity and [*η*] was shown in Figure 3. 

From Figure 3, it was found out that the change of the *η*_a_ of P(DAC-AM) samples decreasing to the lowest value was accelerated by increasing the concentration of multivalent salts of different anions, like Na_2_SO_4_ and Na_3_PO_4_, in solution, and the acceleration was clearly faster even than that of the same multivalent salt of different cations. Similarly, *η*_a_ increased after arriving at the lowest value and the rise rate of *η*_a_ of the P(DAC-AM) samples was enlarged with the increase of the valence of the multivalent salts of different anions.

##### The General Regulations of *η*_a_ of P(DAC-AM) Samples in the Multivalent Salt Solutions

From the above two experimental results and the corresponding Figure 2 and Figure 3, the lowest value of *η*_a_ of a P(DAC-AM) sample shows that the P(DAC-AM) molecules form a tight curl and finally arrive at an unshrinkable state. After that, the phenomena of the increase of *η*_a_ of P(DAC-AM) samples occurred with the increase of salt concentration, only in the divalent salt, such as MgCl_2_ and Na_2_SO_4_, and trivalent salt, like AlCl_3_ and Na_3_PO_4_, solutions. Here, it should be mentioned that these phenomenon were reported by a few studies, however, without a systematic description of the divalent salts [34], and also without a trivalent ion. Meanwhile, the cationicity and [*η*] of P(DAC-AM) and the valence of multivalent salts had the effect on the lowest value of *η*_a_ of P(DAC-AM) sample solutions, extensively, which was similar to the monovalent salts.

### 3.2. The Inflection Point of the η_a_ of P(DAC-AM) Samples in Salt Solutions and Its Influencing Factors

According to the curves in Figure 1 to Figure 3, drawing a horizontal line parallel to the horizontal axis, and then moving it up until its first point of intersection with the curve; this point was the inflection point of the *η*_a_, named *η*_aL_ and the associated salt concentration, named *c*_salt_. Then the influence factors on *η*_aL_ and *c*_salt_ could be accordingly investigated.

#### 3.2.1. The Inflection Point of the η_a_ of P(DAC-AM) Samples and Its Influencing Factors

**Figure 4:**
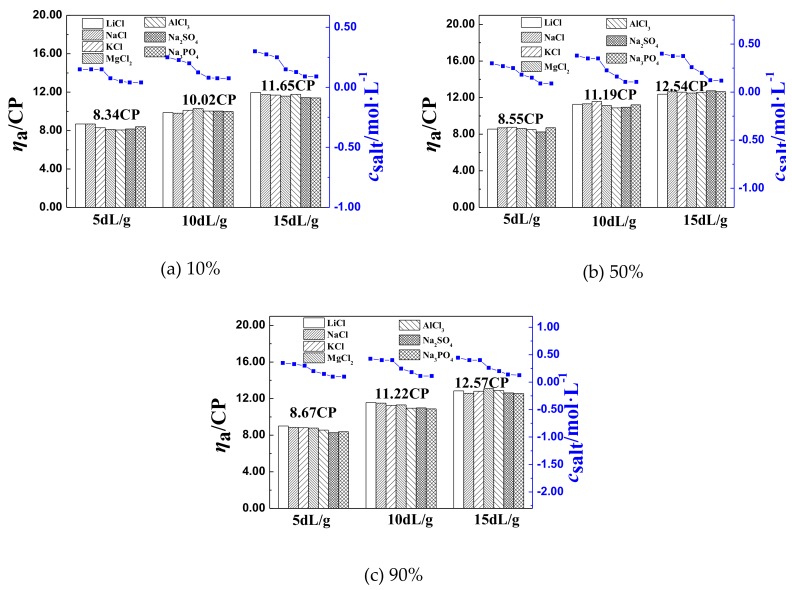
The data of *η*_aL_ and *c*_salt_ of the poly(acryloyloxyethyl trimethyl ammonium chloride–*co*–acrylamide), P(DAC-AM) samples.

The corresponding *c*_salt_ and *η*_aL_ of P(DAC-AM) samples with different cationicity and serial intrinsic viscosity ([*η*]) in the different salt solutions were made via bar graphs and curves, which were shown in Figure 4a–c. Where, the color bar represented the *η*_aL_ of P(DAC-AM) samples in the different salt solutions, the curves with solid dots represented the *c*_salt_ of P(DAC-AM) samples in the different salt solutions.

Figure 4a–c represented the obtained experimental results of using P(DAC-AM) samples with three kinds of cationicity of 10%, 50%, and 90%, respectively. There were the change regulations of the corresponding *c*_salt_ and *η*_aL_ of the P(DAC-AM) with [*η*] of 5, 10, and 15 dL/g of each kind of cationicity in the seven kinds of inorganic salt, such as NaCl, LiCl, KCl, MgCl_2_, AlCl_3_, Na_2_SO_4_ and Na_3_PO_4_, solutions, respectively. In addition, the values above each bar graph represented the average value of the *η*_aL_ of each P(DAC-AM) sample with the same [*η*] and cationicity, but in the different salt solutions.

Firstly, from any of Figure 4a–c, it was observed that the *η*_aL_ of P(DAC-AM) samples with the same cationicity increased with the growing [*η*] in the different salt solutions. Secondly, compared to the *η*_aL_ of P(DAC-AM) samples with the same [*η*] in Figure 4a–c, the *η*_aL_ of P(DAC-AM) samples increased a little with the cationicity increasing, but very slightly. Finally, from the curves of *c*_salt_ above corresponding to *η*_aL_ of the sample with any [*η*] in Figure 4a–c, it was shown that the *c*_salt_ associated with *η*_aL_ of each P(DAC-AM) sample in the different salt solutions were different, which meant the *c*_salt_ was affected by salt kinds and their ion valence. In particular, the *c*_salt_ of all P(DAC-AM) samples decreased with an increase in salt valence.

#### 3.2.2. The Effect of [η] on *c*_salt_ and *η*_aL_ of P(DAC-AM) Samples

##### The Effect of [η] on *η*_aL_

From Section 3.2.1, we demonstrated the effect of [*η*] on both the *η*_aL_ and *c*_salt_ of P(DAC-AM) samples in the salt solutions. From the view of *η*_a_, the *η*_aL_ of P(DAC-AM) samples with the same [*η*] were close each other but a small fluctuation existed in the different salt solutions from Figure 4a–c. Therefore, the average values were summarized in Table 1 for further discussion.

From Table 1, it is shown that the *η*_aL_ of P(DAC-AM) samples of the same cationicity increased from 8.52 to 12.25 CP with the [*η*] increasing from 5 to 15 dL/g. Since the higher [*η*] indicated the higher molecular weight, the minimum volume of polymer after shrinking under the function of salt ions was bigger. This agreed with the research results reported in the literature [30].

##### The Effect of [*η*] on *c*_salt_

Based on the curves of *c*_salt_ and *η*_aL_ of P(DAC-AM) samples in Figure 4, the data of *c*_salt_ of P(DAC-AM) samples with different cationicity and [*η*] in different salt solutions were summarized in Table 2 for further discussion.

In Table 2, it was shown that in the same salt solution, the *c*_salt_, the salt concentration at the inflection point of *η*_a_, increased with the [*η*] of P(DAC-AM) samples with any cationicity. This was because the [*η*] of P(DAC-AM) sample was higher, as well, not only was the volume of P(DAC-AM) molecules larger, but also the total number of the cation functional groups in each molecule increased, resulting in the molecule being more difficult to shrink. Therefore, it was discovered that after comparing with P(DAC-AM) samples of the same kind of cationicity, the *c*_salt_ relating to the molecule shrinking to the minimum volume increased with the molecular weight increasing.

#### 3.2.3. The Effect of Cationicity on c_salt_ and η_aL_ of P(DAC-AM) Samples

From Table 1, the *η*_aL_ of P(DAC-AM) samples of the same [*η*] varied slightly with the cationicity increasing from 10% to 90%, and the relative average deviation of the variation was between 1.41% and 4.87%. The reason is that the molecular weights of P(DAC-AM) with the same [*η*] and different cationicity were close, and the effect of the latter on the size of the minimum volume of the molecules shrinking was not revealed, so the *η*_aL_ did not increase greatly at that time.

However, in contrast, from Table 2, it was shown that the *c*_salt_ of P(DAC-AM) samples of the same [*η*] increased clearly with the cationicity increasing. The reason was that when the cationicity of P(DAC-AM) polymers was higher and their [*η*] was the same, that is to say, the cationic hanging groups of the P(DAC-AM) were more, the total number and density of cation groups in each molecule increased and the repulsive force was larger, resulting in the P(DAC-AM) molecule volume becoming more difficult to shrink under the function of salts. So, it was necessary to require more counter ions to help the *η*_a_ to arrive at their inflection point *η*_aL,_ the minimum volume of the P(DAC-AM) molecule. From all of the above, it was not difficult to find that the minimum volume of the P(DAC-AM) samples with the same [*η*] in the salt solutions changed a little by the increase of cationicity, but the increase of their cationicity had a clear effect on the *c*_salt_. 

#### 3.2.4. The Effect of Salt Ion Valence on *c*_salt_ and *η*_aL_ of the P(DAC-AM) Samples

The influence regulations of the cationicity and [*η*] of P(DAC-AM) samples on the *c*_salt_ and *η*_aL_ were discussed in Section 3.2.2 and Section 3.2.3, respectively. Obviously, from either Figure 4 or Table 2, it is observed that the *η*_aL_s of the same P(DAC-AM) sample in different salt solutions are close, but their corresponding *c*_salt_s are different. Especially, from the data of *c*_salt_ related with the *η*_aL_ of P(DAC-AM) samples in Table 2, it was shown that these effects of the different kinds of salts on the *c*_salt_ of a P(DAC-AM) sample manifested not only as the higher the salt ion valence, the lower the required *c*_salt_, but also that the effect of the anion was larger than that of the cation when they were with the same salt ion valence. Therefore, using any one of P(DAC-AM) samples as an example, e.g. a P(DAC-AM) sample with 50% cationicity and 10 dL/g intrinsic viscosity, and based on the experimental results and summary of Section 3.1 and Table 2, a deep analysis could proceed. Therefore, the *η*_aL_ and *c*_salt_ of the sample in the solution with the same cation and anion salts, such as NaCl, LiCl, KCl, MgCl_2_, AlCl_3_, Na_2_SO, and Na_3_PO_4_, and the corresponding concentration of cation and anion were listed in Table 3 for this purpose.

In Table 3, the different *c*_salt_ values, their corresponding cation concentration (*c*(cation)), and their anion concentration (*c*(anion)) were listed separately, when the *η*_aL_ of the above P(DAC-AM) exampled was 11.19 CP. From the data of Table 3, the conclusions might be acquired as follows.

(1) The effect of the cation radius. For the monovalent salts, like LiCl, NaCl, and KCl in their solutions, with the same anion and different cations, their *c*_salt_ decreased a little with the increase of the cation radius of salts from LiCl to KCl which were in the same group and different period, comparing all corresponding *c*_salt_ at the *η*_aL_ of the P(DAC-AM), as shown for example in Table 3. This might be due to the electronegativity of cations decreasing with their ionic radius increasing, and their ability of ionizing or releasing Cl^−^ increasing [35], which resulted in the increase of the charge shielding effect of free Cl^−^ on the P(DAC-AM) molecules, i.e., the *c*_salt_ at the *η*_aL_ of the P(DAC-AM) exampled decreased.
(2) The effect of cation valence. For the salt, as NaCl, MgCl_2_, and AlCl_3_ in their solutions, with the same anion and different valent cations, their *c*_salt_ decreased with the increase of the cation valence, comparing all corresponding *c*_salt_ at *η*_aL_ of the P(DAC-AM) as shown in Table 3. However, according to the anion and cation concentration, the corresponding cation concentration at the inflection point decreased from 0.350 to 0.162 mol/L with the valence of cation, Na^+^, Mg^2+^, and Al^3+^. However, the corresponding concentration of anion Cl^−^ was 0.350, 0.452, and 0.486 mol/L, which indicated that the concentration of the counter ion, Cl^−^ required to shrink the P(DAC-AM) molecule to the minimum volume in the MgCl_2_ and AlCl_3_ solutions was larger than that in the NaCl solution. Via a comparison with the effect of cation radius in the above (1), the reason might be easily understood that the ability of ionizing or releasing Cl^−^ decreased with both the increase of the nuclear electronic number and the decrease of their ion radius of the cations in the same period, like Na^+^, Mg^2+^, and Al^3+^, which resulted in the decrease of the charge shielding effect of free Cl^−^ on the positive charges in P(DAC-AM) molecules.
(3) The effect of anion valence. For the salt, as NaCl, Na_2_SO_4_, and Na_3_PO_4_ in solutions, with the same cation and different valent anions, their *c*_salt_ decreased clearly with the increase of the anion valence, comparing all the corresponding *c*_salt_ at the *η*_aL_ of the P(DAC-AM) as shown in Table 3. According to the anion and cation concentration, it was clear to see that the *c*_salt_, equal to the anion concentration here as well, decreased sharply from 0.350 to 0.106 mol/L with the valence increase of anions, like Cl^−^, SO_4_^2−^, and PO_4_^3−^, which meant the increase of the charge number of the anions. In this time, however, the corresponding concentration of Na^+^ was 0.350, 0.212, and 0.318 mol/L. This illustrated that the salt cation, Na^+^, had no notable effect on the P(DAC-AM) molecules shrinking, and meanwhile indicated the charge number of the salt anion had a greater effect on the P(DAC-AM) molecules shrinking. The larger the charge number of the anion was, the P(DAC-AM) more easily the molecules shrank. This might be because the multivalent anion could shield more hanging cation groups in P(DAC-AM) molecules than the monovalent anion to make the polymer molecules shrink strongly [19,36].

### 3.3. Mechanism Analysis of Effect of Salt Concentration on the η_a_ of P(DAC-AM) Samples After Inflection Point

From Section 3.1 and Section 3.2, the change regulations of the values of apparent viscosity (*η*_a_) of any one P(DAC-AM) sample in the monovalent and multivalent salt solutions before and at the inflection point were analyzed in detail. However, after the inflection point, the *η*_a_ of any one P(DAC-AM) sample in the monovalent salt solutions was kept unchanged, as when the *η*_a_ arrived at the inflection point, the P(DAC-AM) molecule had been in an unshrinkable state. When the salt concentration increased further, the P(DAC-AM) molecule would still persist in the state at the inflection point and maintain a similar interaction with salt ions [32,33,37]. However, the *η*_a_ of P(DAC-AM) samples in the multivalent salt solutions after the inflection point increased with an increase in the multivalent salt concentration. This phenomena had been simply described in previous studies [34], and had never been analyzed and explained in detail, which will be discussed thoroughly, here.

#### 3.3.1. Speculation and Hypothesis of the Mechanism

From Section 3.1, the *η*_a_ of all P(DAC-AM) samples decreased to their inflection point, and then they increased with the increase of multivalent salt concentration. For these phenomena, it might be speculated that the formation of complexes of multivalent salts themselves resulted in the increase of the *η*_a_ of salt solutions themselves [38,39] or the interaction between the polymers and multivalent salt complexes resulting in the increase of the *η*_a_.

In order to investigate the above possible interaction form which made the *η*_a_ of salt solutions of polymer increase after their inflection point, a hypothesis was first put forward based on the above speculation: First the ionic complexes formed during the increase of concentration of pure salt solutions. The increase of the *η*_a_ of salt solutions resulted from the two interactions simply superimposed, i.e., the interactions, both of these complexes themselves and between these complexes and the P(DAC-AM) molecules. Furthermore, on the basis of the hypothesis, it could be speculated further that if the change of residual values of *η*_a_ of the P(DAC-AM) samples in the multivalent salt solutions, after deducting the *η*_a_ of the corresponding pure salt solutions, remained unchanged (which was similar with the situation in the monovalent salt solutions), it would verify that these increases of *η*_a_ after the inflection point were completely caused by the formation of salt complexes [38,39] and the increase of their concentration. However, if these residual values of *η*_a_ of the P(DAC-AM) samples in the multivalent salt solutions after deducting the *η*_a_ of corresponding pure salt solutions changed or increased, we would discover a new function form, i.e., a novel mechanism of the interaction between the salt complexes and the P(DAC-AM) molecules. Therefore, in order to verify the above speculation and hypothesis, the *η*_a_ of different pure salt solutions was determined, and the change regulations of the residual values of *η*_a_ of P(DAC-AM) samples with salt concentration were investigated after deducting the *η*_a_ of the pure salt solutions tested.

#### 3.3.2. Verifying Experiments and Results

(1) The *η*_a_ of pure salt solutions of different concentrations.

**Figure 5:**
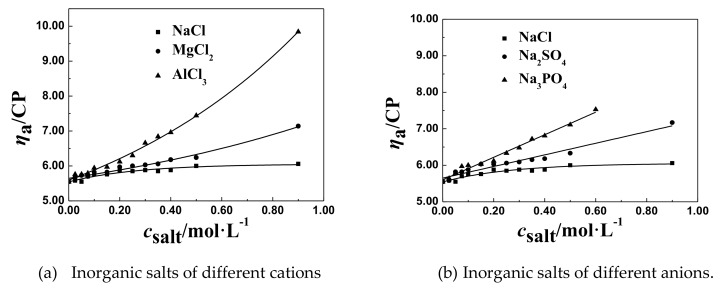
The *η*_a_ of the pure salt solutions.

In order to verify the hypothesis in 3.3.1, the *η*_a_ of the solutions of pure salts, such as NaCl, MgCl_2_, AlCl_3_, Na_2_SO_4_, and Na_3_PO_4_, with different anion and cation valence, chosen from the seven kinds of salts, was determined using a rotational viscometer and the results were shown in Figure 5.

Figure 5 presented the change regulations of the *η*_a_ of pure inorganic salt solutions which increased a little in the lower concentrations with the increase of salt concentration, before and near 0.100 mol/L. After the point of 0.100 mol/L, the changes of the *η*_a_ of different valent salt solutions were divorced. The *η*_a_ of monovalent salt solutions were unchanged or increased a little, but the *η*_a_ of multivalent salt solutions increased linearly with salt concentration. These might verify the formation of complexes in the multivalent salts themselves resulting in the increase of the *η*_a_ of salt solutions [38,39]. This could be the one of the reasons resulting in the increase of the *η*_a_ of P(DAC-AM) samples in multivalent salt solutions. In addition, it was easy to see that from Figure 5, on the one hand, the increasing rate of the *η*_a_ of multivalent salts of different cations with salt concentrations was larger than that of multivalent salts of different anions with the same valence. This might indicate the volume of the complexes formed by the multivalent cation salts themselves was larger than that by the multivalent anion salts, which resulted in the internal resistance between the salt ions increasing more, and indicated the size of the complexes volume was larger, resulting in a greater effect on the *η*_a_ of pure salt solutions. On the other hand, the *η*_a_ curve of the *η*_a_ of AlCl_3_ solutions increased greatly, and with an even larger departure from the linear rule after 0.600 mol/L within the testing range of the salt concentration from 0 to 0.900 mol/L, indicating the existing variety of AlCl_3_ cations in aggregation numbers.

(2) The changes of the residual values of *η*_a_ of P(DAC-AM) after deducting the *η*_a_ of pure salt solutions.

**Figure 6:**
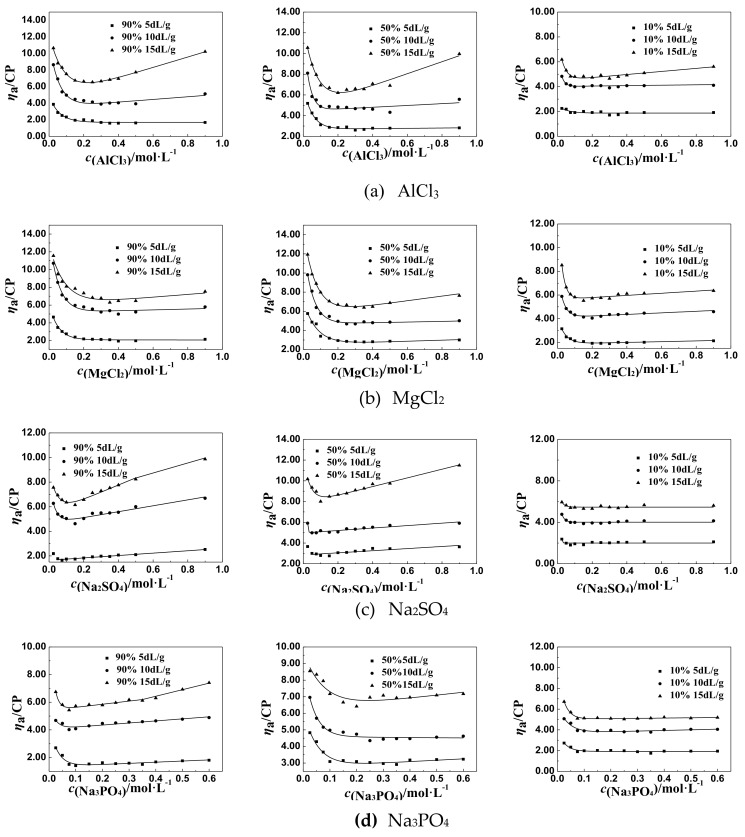
The relationship between the salt concentration and the residual values of *η*_a_ of P(DAC-AM) samples in the multivalent salt solutions.

From the results of the *η*_a_ of pure salt solutions in Figure 5, it was found out the *η*_a_ of pure salt solutions could affect the values of *η*_a_ of P(DAC-AM) samples in the multivalent salt solutions. Therefore, in order to quantitatively investigate the scale of this effect, the changes of the residual values of the *η*_a_ of P(DAC-AM) samples in the multivalent salt solutions after deducting the *η*_a_ of pure salt solutions with the salt concentration were shown in Figure 6. 

Figure 6 showed the curves of residual values of *η*_a_ of P(DAC-AM) samples with cationicity of 10%, 50%, and 90% in the MgCl_2_, AlCl_3_, Na_2_SO_4_, and Na_3_PO_4_ solutions after deducting the *η*_a_ of pure salt solutions as changed with the salt concentration. It was shown that the residual values of *η*_a_ of the P(DAC-AM) samples with cationicity of 10% decreased first and then remained unchanged or experienced a slight increase with the multivalent salt concentration increase. However, the residual values of *η*_a_ of P(DAC-AM) samples with cationicity of 50% and 90% decreased first and then increased with the multivalent salt concentration increasing, and its growth rate increased obviously when after the multivalent salt concentration of 0.400 mol/L for P(DAC-AM) samples with higher molecular weight ([*η*] were 10 and 15 dl/g and it was shown to be a linear relationship with the salt concentration, which illustrated the new interaction form speculated to exist between salt complexes and polymers. Meanwhile, it was seen clearly that in the same salt solution, the growth range of residual values of *η*_a_ of P(DAC-AM) samples after the multivalent salt concentration of 0.400 mol/L increased with the [*η*] and cationicity of polymer samples increasing which meant the larger size of molecule and charge density, respectively. Therefore, the hypothesis was verified that the change of *η*_a_ of polymer salt solutions after the inflection point was caused by not only the *η*_a_ of the salt ion complexes in pure salt solutions [40,41], but also the increased effect of *η*_a_ generated from the interaction between the polymers, P(DAC-AM) molecules and the complexes of salt ions, especially multivalent salt ions. The later was directly affected by the charge density and molecule size of the polymer.

(3) Mechanism analysis and the numerical simulation of the interaction regulation.

According to the verified results in Section 3.3.2 (2), we further quantitatively investigated the increased effect of *η*_a_ generated from the interaction between the polymers and multivalent salt ions or their complexes and corresponding change regulations in the salt solutions by fitting the linear equations of the residual values of *η*_a_ of P(DAC-AM) samples with cationicity of 10%, 50%, and 90% after deducting the *η*_a_ of the pure salt solutions with the salt concentration ranging after the inflection point, for example, from 0.400 to 0.900 mol/L selected. The fitting equations and correlation coefficient (*R*^2^) obtained were shown in Table 4.

In Table 4, the *x* in the fitting equations represented the salt concentration and the unit was mol/L. The *y* represented the residual values of *η*_a_ of P(DAC-AM) samples after deducting the *η*_a_ of pure salt solutions and the unit was CP. The group of entire fitting equations in Table 4 represented the quantitative relationship of the interaction between the salt ion complexes and P(DAC-AM) molecules. From Table 4, for all of the P(DAC-AM) samples, we discovered that the relationship between the residual values of *η*_a_ of salt solutions of P(DAC-AM) samples and the salt concentration obtained by fitting was linear and shown as equations, the *R*^2^s of which, after fitting, were all above 0.9110. Meanwhile, the slopes of the equations were all close to or larger than zero, the intercepts were over zero, as well. The ranges of the values were 0.02–6.45 and 1.45–8.02. These illustrated that the increase of residual values of *η*_a_ of polymers with salt concentration increased linearly from a certain value of *η*_a_ without exception, meanwhile, indicating the unicity of interaction mode between salts and their complexes within the chosen range of salt concentration.

Furthermore, based on the results in Figure 5, Figure 6, and Table 4, it was further observed and analyzed that, on the one hand, for the all salt solutions of P(DAC-AM) samples, it could be conjectured that after the inflection point of *η*_a_ of their salt solutions, the multivalent ions began to form the complexes with a larger volume and similar structure, and only the amount of these complexes increased with the salt concentration increasing. Then these complexes not only caused the increase of the *η*_a_ of salt solutions of polymers with the increase of salt concentration which could be reflected in the changes of the *η*_a_ of pure salt solutions in Figure 5, but also caused the increase of the viscosity resistance with the polymer molecules in the flowing process and made the increase of *η*_a_ again which could be reflected in the changes of the residual values of *η*_a_ of salt solutions of polymers after deducting the corresponding *η*_a_ of pure salt solutions in Figure 6 and Table 4. Therefore, it was really acceptable that the essential reason for the increase of *η*_a_ of salt solutions of P(DAC-AM) samples after their inflection point came from the possible formation of the salt ion complexes.

On the other hand, it could be speculated that the increase of the [*η*] of P(DAC-AM) samples of the same cationicity, meaning the increase of volume size of the molecules, obviously increased the strength of the interaction of the space or volume between salt ion complexes and polymer molecules. Similarly, for the P(DAC-AM) samples with the same [*η*], when the cationicity of the polymer increased, the charge density increased and the charge interaction between the polymers and salt ion complexes strengthened. These two kinds of interactions resulted in the increase of viscosity resistance of polymer solutions in the flowing process, which could be reflected not only from the increase of the data of slopes of linear equations in Table 4 with the increase of cationicity and [*η*] of P(DAC-AM) samples, but also, from all of these being consistent with the nature of the *η*_a_, the rationale of expression of the fricative viscosity resistance in the flowing process of the fluid molecules or particles [27]. Therefore, the volume size and charge density of the P(DAC-AM) molecules had the direct effect on the scale of the viscosity resistance between them and salt ion complexes, which meant fairly the scale of the *η*_a_ of solutions.

### 3.4. Model of the Interaction Mechanism between Salt Ions and Polymer P(DAC-AM)

#### 3.4.1. Model of the Interaction Mechanism Before and at the Inflection Point of *η*_a_

**Figure 7:**
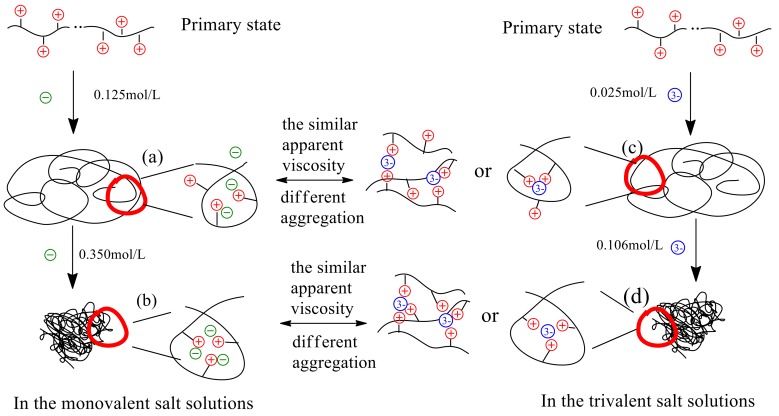
The aggregation models of a polymer P(DAC-AM) sample in monovalent and multivalent salt solutions before and at the inflection point.

Based on the results and discussion in Section 3.1 and Section 3.2, the values of apparent viscosity (*η*_a_) of polymer P(DAC-AM) samples in the salt solutions before the inflection point decreased sharply with the salt concentration increasing, and the salt concentration required to arrive at the same *η*_a_ of any P(DAC-AM) in the different salt solutions was different. The reason should be that the interaction mechanism between the different kinds of salt ions and the polymers was different, which meant that the aggregation models of polymer molecules shrinking made by the salt ion valence and charge were different. Therefore, in order to preferably describe the interactions, i.e., the aggregation states between the P(DAC-AM) samples and salt ions with a different valence, especially the anions, a simple model of the progresses of the decrease of *η*_a_ of the P(DAC-AM) samples and arriving at the inflection point were conjectured and drawn in Figure 7, accompanying the results of the salt concentration required to arrive at the same *η*_a_ (the similar degree of the molecule curl) of the same P(DAC-AM) sample with the same cationicity and intrinsic viscosity ([*η*]) (taking the P(DAC-AM) with cationicity of 50% and [*η*] of 10 dL/g as the example) in the monovalent (anion) and multivalent (trivalent anion) salt solutions.

From Figure 7, it was shown that the whole description of the model was divided into two parts which all had two kinds of shrinking states. One was (a) and (b), it described the P(DAC-AM) molecules from stretching to shrinking in the salt solutions, firstly presented the loose curl and then became the tight curl to arrive at the minimum volume with the monovalent salt concentration increasing from 0 to 0.350 mol/L (the concentration of cation and anion was the same and the value was 0.350 mol/L, for example, NaCl). The other was (c) and (d), it also described the P(DAC-AM) molecules from stretching to shrinking in the salt solutions firstly presented the loose curl or stereo-structure, and then became the tight curl or steric network-like structure to arrive at the minimum volume with the trivalent anion salt concentration increasing merely from 0 to 0.106 mol/L (the corresponding cation concentration was 0.318 mol/L, for example, Na_3_PO_4_). The *η*_a_ of the two proceeding and states in (a) and (c), and (b) and (d) were almost the same, but the salt concentrations required were remarkably different due to the different salt ion valence. The former changed from the primary state to (a) and (c) states, the salt concentration changed from 0 to 0.125 and 0.025 mol/L, respectively. The latter changed from the primary state to (b) and (d) states, the salt concentration changed from 0 to 0.350 and 0.106 mol/L, respectively. The microstructures of the corresponding aggregation states were also different. The reasons for these were that the monovalent anion might only shield one positive charge group, and the trivalent anion might theoretically shield three positive charge groups at the same time [22], so the trivalent anion salt concentration required to arrive at the same *η*_a_ was lower than the monovalent salt concentration due to the different microstructures of the aggregations states.

#### 3.4.2. Model of the Interaction Mechanism After the Inflection Point of the *η*_a_

**Figure 8:**
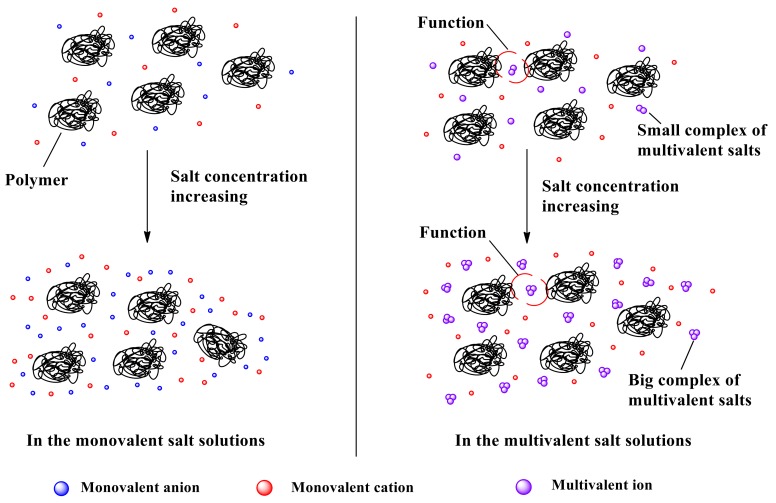
The aggregation model of the polymer P(DAC-AM) samples in salt solutions after the inflection point.

Analogously, based on the mechanism analysis of Section 3.3, after passing the inflection point, the *η*_a_ of the polymer P(DAC-AM) samples remained unchanged with the increase of the monovalent salt concentration, however, they increased with the multivalent salt concentration increasing. This illustrated that the increase of the monovalent salt concentration had no obvious effect on the *η*_a_ of P(DAC-AM) after passing the inflection point, which indicated no new action from the salt complex was formed with an increase of salt concentration after the inflection point. The interaction between the polymers and salt ions continued to maintain the states of arriving at the inflection point within a range of the increase of the salt concentration. However, the increase of the multivalent salt concentration had a great effect on the *η*_a_ of P(DAC-AM) after passing the inflection point which indicated the strength of the interaction between the complexes themselves formed from multivalent salt ions and between these complexes and the polymer molecules increased with the salt concentration increasing. The corresponding model was conjectured and shown in Figure 8.

Figure 8 described the interaction both between the salt ions and their complexes formed by themselves and between the complexes and the polymers in the monovalent and multivalent salt solutions after passing the inflection point of *η*_a_ of the P(DAC-AM) samples.

In the monovalent salt solutions, the P(DAC-AM) molecule had been an unshrinkable curl and the salt ions were free in the independent status, even near the inflection point. A big differentia of the volume between the P(DAC-AM) molecules and salt ions could be seen. The salt ions just existed as the solvent of the polymer, and the *η*_a_ of P(DAC-AM) changed very little with the salt concentration increasing. That was to say that the *η*_a_ of P(DAC-AM) after the inflection point was unchanged in the monovalent salt solutions within a certain range of salt concentration due to the almost unchanged interaction form between the P(DAC-AM) molecules and salt ions. 

However, in the multivalent salt solutions, once the salt concentration passed the concentration point of the inflection point of *η*_a_, the P(DAC-AM) molecules also had an unshrinkable curl. However, at this point the association between the multivalent salt molecules had occurred and they started or had formed the complexes. The complexes should have a larger volume in comparison with their precursor, the independent salt ions. Moreover, when the salt concentration increased further, the complexes’ concentration also increased synchronously, and the interaction both, among the salt ion complexes [41] similar with that in the pure salt solutions, and between the complexes and the P(DAC-AM) molecules, could result in the significant increase of internal flow resistance. That was to say that the *η*_a_ of P(DAC-AM) samples after passing their inflection point still increased distinctly in the multivalent salt solutions.

We should pay particular attention to the increase of the *η*_a_ of salt solutions of polymer, resulting from the salt ion valence, the cationicity, and the [*η*] of the polymer sharing their effect on this interaction between the salt ion complexes and P(DAC-AM) molecules, after passing the inflection point. That is to say that, the increase of the multivalent salt ion valence meant that the complexes volume increased when with similar aggregation numbers, and the increase of the cationicity and the [*η*] of the polymer meant the charge number and molecule size of a P(DAC-AM) molecule increased respectively. They had ultimately enhanced the interaction between the salt ion complexes and P(DAC-AM) molecules after the inflection point, i.e., the performance of the increase of *η*_a,_ in different modes and degrees.

## 4. Conclusions

(1) The values of apparent viscosity (*η*_a_) of P(DAC-AM) with cationicity of 10%, 50%, and 90%, and intrinsic viscosity ([*η*]) of 5, 10, and 15 dL/g in the inorganic salt, such as NaCl, LiCl, KCl, MgCl_2_, AlCl_3_, Na_2_SO_4_, and Na_3_PO_4_, solutions firstly decreased sharply to their inflection point with the salt concentration, then tended to maintain their values with the increase of the monovalent salt concentration, or increased with the increase of the multivalent salt concentration after the inflection point.
(2) It was discovered that the [*η*], cationicity, the kinds of salts, and the salt valence shared in the effect on the values of apparent viscosity (*η*_aL_) at the inflection point of *η*_a_ of P(DAC-AM) samples, and corresponding salt concentrations (*c*_salt_). For the polymer P(DAC-AM), samples of the same cationicity, the *c*_salt_ and *η*_aL_ increased with the [*η*] of P(DAC-AM) increasing. For the polymer P(DAC-AM) samples of the same [*η*], the *c*_salt_ increased with the cationicity increasing, however, the cationicity of the polymer P(DAC-AM) samples hardly impacted the *η*_aL_. For the same P(DAC-AM) sample, the *c*_salt_ required to arrive at the inflection point of the *η*_a_ of P(DAC-AM) decreased with the increase of the cation or anion valence in the salt solution, and here, it decreased lower with the anion valence in the salt solutions.
(3) From the mechanism hypothesis and verifying experiments, we discovered that the reasons for the increase of the *η*_a_ of P(DAC-AM) samples in the multivalent salt solutions after passing the inflection point were caused by not only the increase of *η*_a_ of the pure salt solutions resulting from the formation of the salt ion complexes, but also the interaction between the polymers and salt ion complexes. The linear quantitative relationship of the latter was developed and obtained. Meanwhile, we recognized that an increase in both the multivalent salt ion valences (meaning the volume and charge of the complexes) and the cationicity and [*η*] of the P(DAC-AM) samples (meaning the charge and the molecular size) were all enhanced in these interactions, in their individual form, and to different degrees.
(4) Taking the monovalent ion and trivalent ion as examples, a mechanism model of the interaction between the P(DAC-AM) molecules and inorganic salt ions or their complexes was set, in order to illustrate the entire progress from the decrease of the *η*_a_ of P(DAC-AM) samples to their inflection point and after passing through the inflection point in the salt solutions. On the basis of the model, the dependence of the *η*_a_ of P(DAC-AM) samples on both the cationicity and [*η*] themselves, and the different kinds and valence of salt solutions were described and explained.

## Figures and Tables

**Table 1:** *η*_aL_ of P(DAC-AM) with serial cationicity and [*η*].

No.	[*η*]/dL·g^−1^	*η*_aL_/CP				Relative Average Deviation/%
		10%	50%	90%	Average *η*_a_	
1	5	8.34	8.55	8.67	8.52	1.41
2	10	10.02	11.19	11.22	10.81	4.87
3	15	11.65	12.54	12.57	12.25	3.29

**Table 2:** The *c*_salt_ of P(DAC-AM) with serial cationicity and [*η*].

[*η*]/ dL·g^−1^	Cationicity/ %	*c*_salt_/ (mol•L^−1^)						
		LiCl	NaCl	KCl	MgCl_2_	AlCl_3_	Na_2_SO_4_	Na_3_PO_4_
5	10	0.150	0.150	0.150	0.075	0.050	0.040	0.040
	50	0.300	0.270	0.250	0.182	0.150	0.090	0.090
	90	0.350	0.328	0.300	0.200	0.150	0.100	0.100
10	10	0.250	0.227	0.200	0.124	0.080	0.075	0.075
	50	0.380	0.350	0.350	0.226	0.162	0.106	0.106
	90	0.425	0.400	0.400	0.247	0.182	0.112	0.112
15	10	0.300	0.274	0.250	0.150	0.127	0.090	0.090
	50	0.400	0.375	0.375	0.260	0.200	0.124	0.119
	90	0.445	0.400	0.400	0.260	0.200	0.139	0.127

**Table 3:** The *η*_aL_ and *c*_salt_ of a P(DAC-AM) exampled in the salt solutions and the corresponding concentration of cation and anion.

Salt Names	*η*_aL_ = 11.19 CP		
	*c*_salt_/(mol·L^−1^)	*c*(cation)/(mol·L^−1^)	*c*(anion)/(mol·L^−1^)
LiCl	0.380	0.380	0.380
NaCl	0.350	0.350	0.350
KCl	0.350	0.350	0.350
MgCl_2_	0.226	0.226 (<0.350)	0.452 (>0.350)
AlCl_3_	0.162	0.162 (<0.350)	0.486 (>0.350)
Na_2_SO_4_	0.106	0.212 (<0.350)	0.106 (<0.350)
Na_3_PO_4_	0.106	0.318 (<0.350)	0.106 (<0.350)

**Table 4:** The fitting equations and *R*^2^ of residual values of *η*_a_ of P(DAC-AM) samples in the multivalent salt solutions.

Salts	[*η*]/ (dL·g^−1^)	The Fitted Equations					
		90%	*R* ^2^	50%	*R* ^2^	10%	*R* ^2^
AlCl_3_	5	*y* = 0.17*x* + 1.53	0.9547	*y* = 0.06*x* + 2.76	0.9901	*y* = 0.06*x* + 1.90	0.9592
	10	*y* = 2.19*x* + 3.11	0.9618	*y* = 1.95*x* + 3.82	0.9960	*y* = 0.06*x* + 4.05	0.9286
	15	*y* = 6.45*x* + 4.44	0.9968	*y* = 6.02*x* + 4.50	0.9691	*y* = 1.35*x* + 4.40	0.9918
MgCl_2_	5	*y* = 0.41*x* + 1.78	0.9949	*y* = 0.36*x* + 2.67	0.9695	*y* = 0.30*x* + 1.86	1.0000
	10	*y* = 1.56*x* + 4.39	0.9791	*y* = 0.41*x* + 4.65	0.9835	*y* = 0.34*x* + 4.28	0.9592
	15	*y* = 2.25*x* + 5.48	0.9286	*y* = 2.17*x* + 5.69	0.9495	*y* = 0.49*x* + 5.91	0.9887
Na_2_SO_4_	5	*y* = 0.94*x* + 1.66	0.9662	*y* = 0.47*x* + 3.21	0.9945	*y* = 0.08*x* + 2.04	0.9945
	10	*y* = 2.12*x* + 4.81	0.9110	*y* = 0.75*x* + 5.24	0.9737	*y* = 0.06*x* + 4.09	0.9592
	15	*y* = 4.18*x* + 6.12	0.9984	*y* = 3.81*x* + 8.02	0.9488	*y* = 0.39*x* + 5.35	0.9235
Na_3_PO_4_	5	*y* = 0.60*x* + 1.45	0.9286	*y* = 0.25*x* + 3.08	0.9737	*y* = 0.10*x* + 1.90	1.0000
	10	*y* = 1.20*x* + 4.17	1.0000	*y* = 0.75*x* + 4.18	0.9737	*y* = 0.15*x* + 3.96	0.9286
	15	*y* = 5.55*x* + 4.11	0.9879	*y* = 1.05*x* + 6.55	0.9629	*y* = 0.30*x* + 5.01	0.9286
